# The Impact of Triglyceride-Glucose (TyG) Index on Future Cardio and Cerebrovascular Events in Patients with Acute Coronary Syndrome, During 3 Years of Follow-Up

**DOI:** 10.3390/jcdd11110354

**Published:** 2024-11-05

**Authors:** Francesca Macaione, Daniela Di Lisi, Cristina Madaudo, Alessandro D’agostino, Daniele Adorno, Vincenzo Sucato, Giuseppina Novo, Salvatore Evola

**Affiliations:** 1 Division of Cardiology, University Hospital Paolo Giaccone, 90127 Palermo, Italy; daniela.dilisi@policlinico.pa.it (D.D.L.); alessandro.dagostino80@policlinico.pa.it (A.D.); danieleadorno@policlinico.pa.it (D.A.); salvatore.evola@policlinico.pa.it (S.E.); 2 Department of Health Promotion, Mother and Child Care, Internal Medicine and Medical Specialties (PROMISE) “G. D’Alessandro”, University of Palermo, 90127 Palermo, Italy; cristina.madaudo@gmail.com (C.M.); vincenzo.sucato@policlinico.pa.it (V.S.); giuseppina.novo@policlinico.pa.it (G.N.)

**Keywords:** triglyceride-glucose index, cardiovascular disease, acute coronary syndrome, insulin resistance, HOMA index

## Abstract

**Background:** The triglyceride-glucose (TyG) index is a new alternative insulin resistance (IR) biomarker. The purpose of this study was to assess whether the TyG index can have a prognostic value in patients with acute coronary syndrome (ACS). Moreover, we wanted to compare the TyG index with HOMA index. **Methods:** We retrospectively enrolled 115 consecutive subjects, 81 males and 34 females, referred for ACS to our Unit of Cardiovascular Care of Policlinico Paolo Giaccone, Palermo. The subjects were divided into tertiles according to TyG index values and we performed a 3-year follow-up study. We considered as an end point new cardiovascular and cerebral events (MACCEs) during follow-up. **Results**: We found a significant statistical correlation between the HOMA index and the TyG index (*p* = 0.001). Patients with elevated TyG index have a higher incidence of MACCE at a 3-year follow-up. In our study the TyG index was an independent predictor of MACCEs (95% CI 1.8158 to 16.8068; P 0.0026) and the optimal TyG index cut-off for predicting MACCEs was 4.92 (sensitivity 76.56% and specificity 72.55%). **Conclusions:** The TyG index seems to significantly have an important prognostic role in patients with ACS and high values of TyG index are superior to HOMA-IR in predicting MACCEs.

## 1. Introduction

Acute coronary syndrome is the primary cause of mortality and disability in industrialized countries [1]. The cumulative mortality of acute coronary syndrome (ACS) patients after 5 years is about 20% [2].

Therefore, the prevention of major adverse cardiovascular events (MACEs) through the reduction of risk factors has important implications for public health and clinical practice.

Insulin resistance (IR) is a critical mechanism of the pathogenesis of diabetes mellitus.

Several studies have demonstrated that IR is a potential independent risk factor for the development of cardiovascular disease (CVD) [3]. It follows that the early detection of IR may contribute to the prevention of CVD.

A hyper-insulinemic euglycemic clamp is considered the gold standard to assess IR but is a laborious, expensive and complex method.

To date, the most popular index to evaluate IR is the homeostasis model assessment of insulin resistance (HOMA-IR), based on fasting glucose and insulin [4,5]. However, the lack of standardization of insulin immunoassays makes the HOMA index less accurate [6]; moreover, circulating insulin concentrations are not routinely measured in primary care settings, which renders HOMA-IR inappropriate for large-scale studies [7].

The triglyceride-glucose (TyG) index, which is the logarithmized product of fasting triglyceride (TG) (mg/dL) and glycemia (mg/dL), has been shown to be a simple alternative method to evaluate insulin resistance [8]. Furthermore, this index has been shown to highly correlate with the hyperinsulinemic-euglycemic clamp and HOMA-IR [9,10]. Previous studies have shown that TyG index is significantly related to an increased risk of cardiovascular events [11,12,13,14].

The aim of our study was to assess whether the TyG index can have a prognostic value in patients for cardiovascular and cerebrovascular events (MACCEs) in patients with ACS. Moreover, we wanted to compare the TyG index with HOMA index.

## 2. Methods

We retrospectively enrolled 115 consecutive subjects, 81 males and 34 females, referred for ACS, to our Unit of Cardiovascular Care of Policlinico Paolo Giaccone, Palermo, Italy, between 2017 and 2019.

ACS includes unstable angina pectoris (UAP), non-ST-elevation myocardial infarction (NSTEMI) and ST-elevation myocardial infarction (STEMI), in accordance with the guidelines of European Society of Cardiology [15].

The project design included a medical examination, biochemical analyses and instrumental exams such as echocardiography, coronary angiography and ecocolordoppler of carotid arteries.

Subjects were excluded from the study if they had type I diabetes, acute inflammatory diseases, hepatic or renal failure, disreactive disorders, autoimmunity and cancer.

HOMA index was calculated as insulin (IU/mL) × glycaemia (mmol/L)/22.5, by dosing fasting and post-prandial glycaemia and insulin [16]. Since insulin therapy or oral diabetes medications could influence serum insulin levels, patients had to stop them at least 1 day before the blood sample. The TyG index was calculated as the fasting triglyceride (mg/dL)  ×  fasting plasma glucose (FPG) (mg/dL)/2.

Renal function was assessed using the CKD-EPI (Chronic Kidney Disease Epidemiology Collaboration) Equations for Glomerular Filtration Rate (GFR) [17].

Heart failure (HF) is commonly classified based on left ventricular ejection fraction (LVEF), so we used echocardiography to individuate HF with preserved (HFpEF; LVEF  ≥  50%), mid-range (HFmrEF; LVEF 40–49%) and reduced ejection fraction (HFrEF; LVEF  <  40%) [18,19]. We also evaluated arterial wall thickness in the carotid arteries using Ecocolor Doppler Examination, statin therapy and medications with antiplatelet drugs at admission to hospital.

Subjects were divided into three groups, according to the distribution in tertiles of the TYG index values.

We performed a three-year follow-up by telephone or outpatient clinical visit, to estimate the incidence of new MACCEs, as Angina Pectoris, Acute Myocardial Infarction or Reacute Myocardial Infarction, cardiac failure, coronary revascularization with CABG (Coronary Artery Bypass Grafting) or PTCI (Percutaneous Transluminal Coronary Intervention) and stroke.

**Figure 1:**
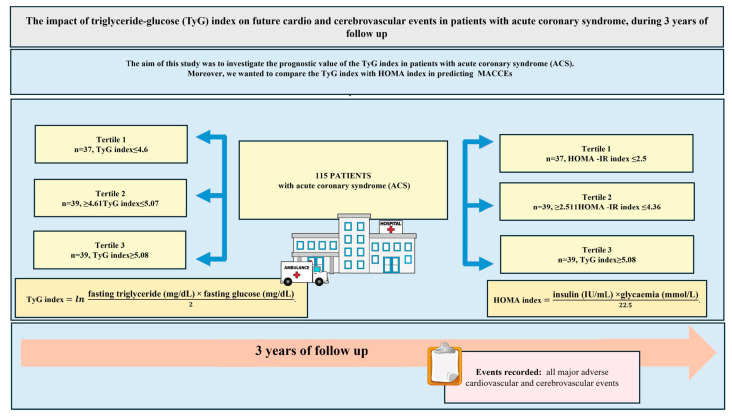
Central illustration. Graphical Presentation of the Study Design, Main Results and Conclusion. ACS, Acute Coronary Syndrome, HOMA index, homeostasis model assessment-insulin resistance; MACCE, major adverse cardiovascular and cerobrovascular events; TyG index, triglyceride-glucose index.

This study was approved by the local research ethics committee and strictly adhered to the Declaration of Helsinki. The study design is presented in the central illustration (Figure 1).

### Statistical Analysis

Baseline characteristics of study participants are described across TyG index variability tertiles.

Continuous variables are expressed as means ± SD or median and inter-quartile range (IQR) according to the variable distribution; binary variables are expressed as counts and percentages.

Comparison among groups was achieved by using one-way ANOVA or Kruskal–Wallis test.

Stepwise multivariable linear regression models were employed to individuate independent predictor of MACCEs.

A comparison of receiver operating characteristic (ROC) curves was used to evaluate sensitivity and specificity of quantitative parameters that multivariate analysis resulted as being statistically significant to indicate its predictive value for MACCEs.

Moreover, the Cox proportional-hazard model was used to test the association between the considered prognostic variables and the outcomes. All variables showing an association with *p* < 0.10 at the univariate model were placed in the multivariate model and were ruled out only if they did not significantly improve the adjustment of the model. The results were presented as hazard ratio (HR) with 95% confidence intervals (CI). Kaplan–Meier curves were generated by relating the development of an outcome over time to each significant prognosticator. The log-rank test was used to compare different strata in Kaplan–Meier analyses.

A two-sided analysis with a *p* value < 0.05 was considered significant. All of the analyses were performed using Statistical Package for Social Science version 22.0 (SPSS Inc., Chicago, IL, USA) and for Windows version 7.2.1.0 (MedCalc Software, Mariakerke, Belgium) statistical packages.

## 3. Results

The baseline clinical characteristics of 115 enrolled patients are listed in Table 1. The study patients had an average age of 65.71 ± 12.17 years and 81 (70.43%) patients were male. The patients were divided into tertiles according to their admission TyG index levels: tertile 1 (n = 37, TyG index ≤ 4.6), tertile 2 (n = 39, ≥4.61 TyG index ≤ 5.07) and tertile 3 (n = 39, TyG index ≥ 5.08). The mean levels of TyG index of the three groups were 4.52 ± 0.16, 4.96 ± 0.06 and 5.30 ± 0.12 respectively (*p* < 0.001).

There were significant differences (*p* < 0.05) among the three groups in terms of diabetes (*p* < 0.001), normal glucose tolerance (NGT) (*p* = 0.002), impaired glucose tolerance (IGT) (*p* = 0.015), creatinine (*p* = 0.020), triglycerides (*p* < 0.001), fasting glucose (*p* < 0.001), post-prandial glucose (*p* = 0.006), post-prandial insulin (*p* = 0.006) and HOMA-IR index (*p* = 0.032).

**Figure 2:**
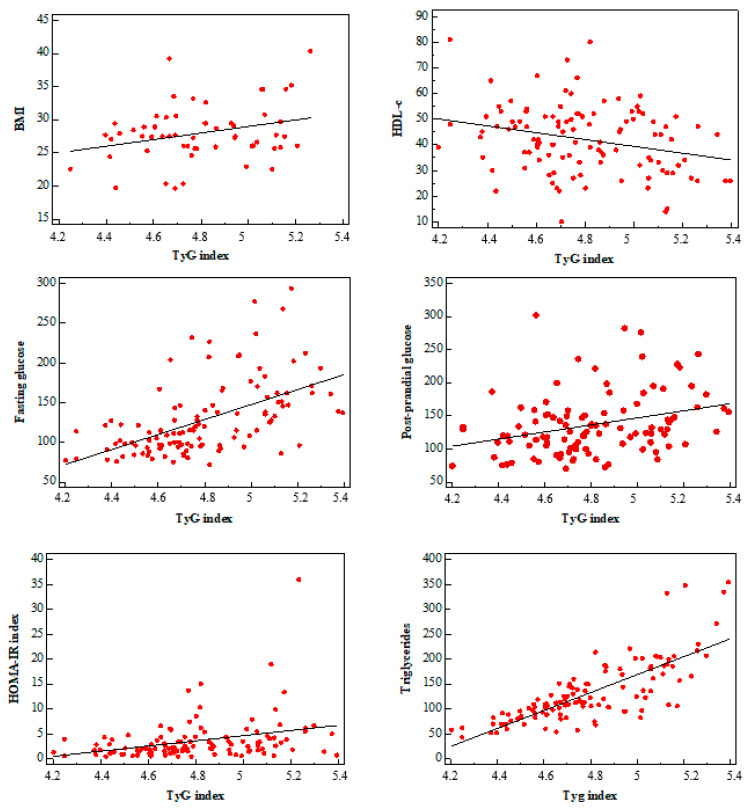
Associations between the TyG index and cardiovascular risk factors (TyG index and HOMA index, triglycerides, HDL-C, BMI, fasting glucose and post-prandial glucose).

The associations between the TyG index and cardiovascular risk factors were evaluated by linear regression analysis. As shown in Figure 2, TyG index levels were positively associated with the HOMA IR index (*p* = 0.001), BMI (*p* = 0.027), diabetes (*p* < 0.001), triglycerides (*p* < 0.001), fasting glucose (*p* < 0.001) and post-prandial glucose (*p* = 0.001), and were negatively associated with col HDL (*p* = 0.0037).

### Follow-Up

During the three-year follow-up, clinical cardiovascular and cerebrovascular events data were fully recorded for 115 patients. During this period, 63 (54.75%) MACCEs were recorded, including 31 (27.67%) unstable anginas, 11 (9.56%) acute myocardial infarctions, 9 (7.82%) heart failures, 9 (7.82%) cardiac arrests, 19 (16.52%) coronary revascularizations and 3 (2.60%) strokes. Every cardio and cerebrovascular event of the study participants are described, across TyG tertiles, in Table 2.

At three-year follow-up, patients with elevated TyG index had a statistically significant incidence of MACCEs compared with patients belonging to the first tertile (*p* < 0.001). Specifically, among MACCEs, there were significant differences among the three groups in terms of unstable angina (*p* = 0.01).

Univariable logistic regression analysis indicated that diabetes, fasting glucose, fasting insulin and HOMA-IR index BMI were statistically associated with MACCEs.

**Figure 3:**
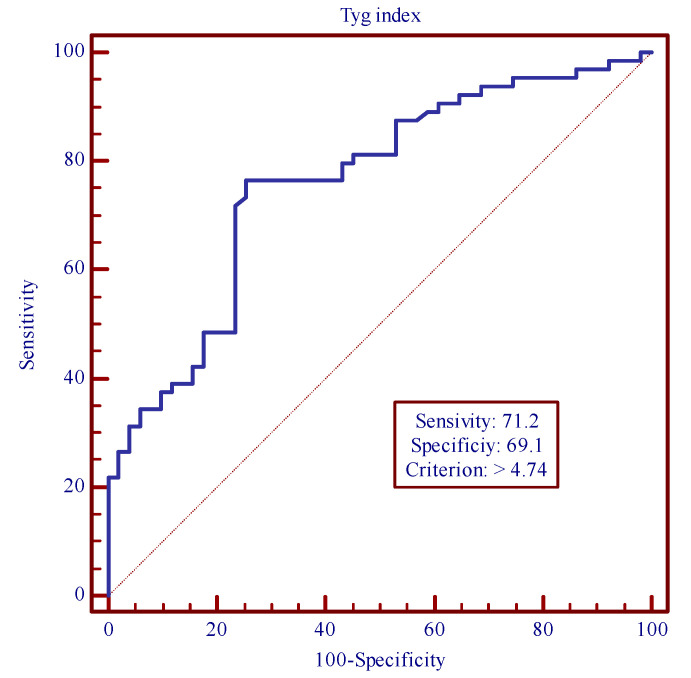
ROC curve to estimate the diagnostic value of TyG index level for predicting MACCEs. MACCEs (major adverse cardiovascular and cerebrovascular events).

Stepwise multivariable logistic regression analysis represented the TyG index as the only independent predictor of MACCEs [odds ratio 3.75; 95% confidence interval (CI) 1.0950 to 12.8421; *p* = 0.032] after adjusting for clinical, functional, bio humoral and echocardiographic parameters. In the stepwise multivariable logistic regression analysis, we included sex, age, mean age, diabetes, hypercholesterolaemia, BMI, NGT, IFG, NSTEMI, STEMI, UAP, number of vessels treated, creatinine, GFR, previous cardiovascular events, total cholesterol, LDL-C, HDL-C, triglycerides. mg/dl, Hs-CRP, ESR, mean TyG index, uric acid, fibrinogen, troponinI, FPG, PPG, fasting insulin, post-prandial insulin, HOMA-IR, HbAlc, LVEF < 40%, LVEF 40–49%, LVEF ≥ 50% and medications at discharge. The ROC curve was used to estimate the diagnostic value of the TyG index level for predicting MACCEs. The ROC analysis showed that the optimal cut off value of the TyG index level for predicting MACCEs was 4.74 (sensitivity 71.2% and specificity 69.1%), with an area under the curve (AUC) of 0.760 (95% CI: 0.672 to 0.835, *p* = 0.0001) (Figure 3).

In unadjusted Cox modeling, only the rate of MACCEs rose significantly with elevated TyG index levels (*p* = 0.01 for trend) and HOMA-IR index (*p* = 0.03 for trend).

Multiple regression analysis, including the emerged predictors and other well-known potential confounders, were performed to assess independent association with LVEF change. Variables to include in the multivariate analysis were chosen based on biological plausibility and were included as a block. Then, a stepwise model was used as a confirmatory analysis.

In a multivariate-adjusted hazard ratio (HR), including the predictors and other well-known potential confounders, the TyG index was found to be an independent predictor (*p* = 0.002).

Age, sex, smoking, BMI, LVEF, diabetes, hypertension, obesity, fasting glucose, fasting insulin, the HOMA-IR index, hs-CRP, statin use and insulin use were included in the multivariate analysis.

For a better comparison between the TyG index and the HOMA-IR index, the patients were divided into tertiles according to their admission HOMA-IR index levels: tertile 1 (n = 37, HOMA-IR index ≤ 2.50), tertile 2 (n = 39, ≥2.51 HOMA-IR index ≤ 4.36) and tertile 3 (n = 39, HOMA-IR index ≥ 4.38).

**Figure 4:**
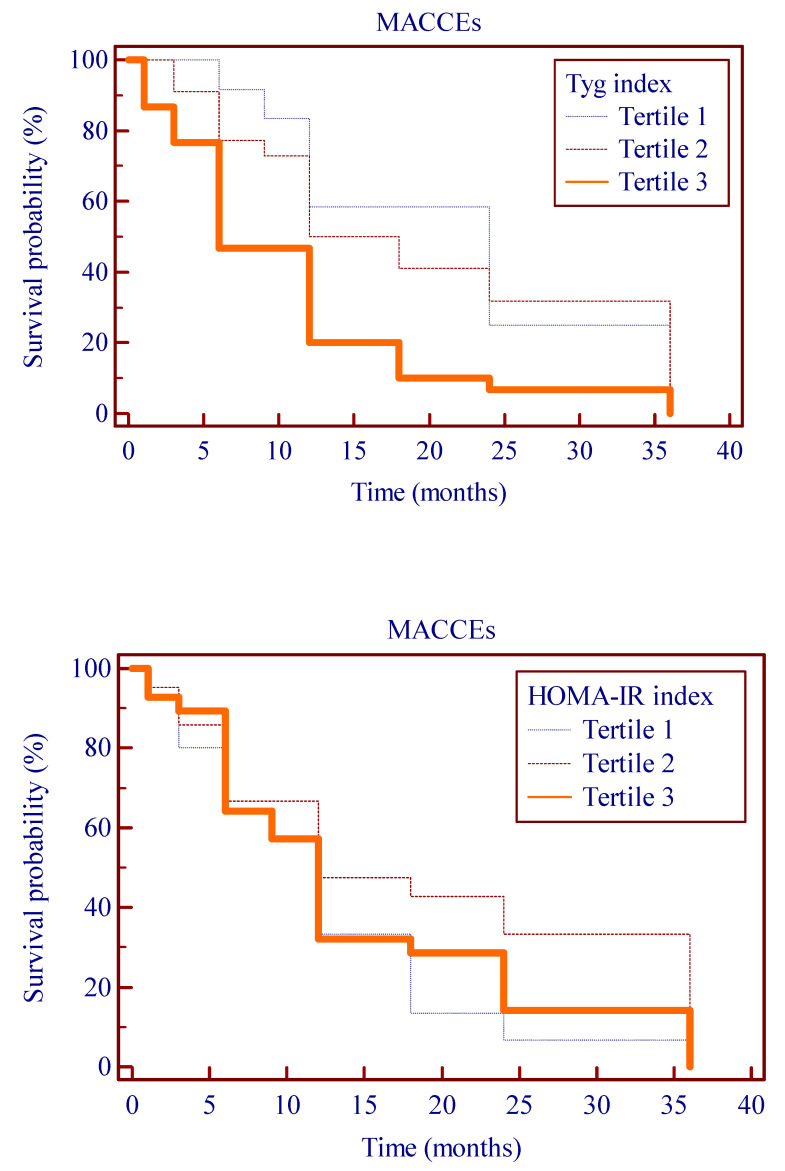
Kaplan–Meier survival curve for MACCE across TyG index tertiles and across HOMA index tertiles. HOMA index, homeostasis model assessment-insulin resistance; MACCE, major adverse cardiovascular and cerobrovascular events; TyG index, triglyceride-glucose index.

As shown in Figure 4, Kaplan–Meier survival analysis showed that the cumulative incidence of MACCEs increased with higher tertiles of the TyG index (log-rank test, *p* = 0.0282).

## 4. Discussion

The present study provides three main findings:

(1) Patients with a higher TyG index had a significantly increased risk of developing cardiovascular events.
(2) The association between TyG index and MACCEs is independent of traditional cardiovascular risk factors and other well-known potential con founders. It follows that the TyG index could be an additional marker for cardiovascular risk assessment.
(3) Both TyG index and HOMA-IR had a relationship with MACCEs, but high values of TyG index are superior to HOMA-IR in predicting MACCEs.

In the last years, several studies showed that the TyG index was an independent risk factor for CVD; however, it must be emphasized that many of these studies did not enroll participants from specific high-risk groups but from the general population [14,20]. In our study, we evaluated the prognostic role of the TyG index to predict MACCEs in patients with ACS, patients with a high risk of developing future cardiovascular and cerebrovascular events.

In the multivariate analysis, important variables lose their power in predicting MACCEs and the only predictor positively associated with increased MACCEs was the TyG index, revealing the prognostic value of this index. Moreover, the ROC curve showed a satisfactory diagnostic value of the TyG index level, equal to 4.74 (sensitivity 71.2% and specificity 69.1%) for predicting MACCEs. It is important to note that the TyG index cut-off proposed in this study is lower than can be used clinically and, despite this, it has shown an important prognostic impact [21]. Consequently, lowering the TyG index level could improve the clinical prognosis of patients with ACS.

A previous study of Wang et al. showed an association between the TyG index and adverse cardiovascular events in patients with diabetes and ACS independently of known cardiovascular risk factors, suggesting that the TyG index may be a useful marker for risk stratification and prognosis in patients with diabetes and ACS [22]. A limitation of this study was that it did not include the HOMA-IR index; therefore, a comparison of the predictive value of the TyG index and HOMA-IR was not explored. Our study is one of the few studies that compared the TyG index with the HOMA-IR index. We found a significant statistical correlation between higher HOMA-IR index values and high levels of TyG index. However, after adjusting for confounding factors, the TyG index was superior to HOMA-IR for predicting MACCEs. Our results were in line with previous studies that have suggested that the TyG index, as the product of FPG and triglycerides and a reliable surrogate of IR, was better than the HOMA-IR index in predicting cardiovascular events [23,24], the development of atherosclerosis and poor outcomes such as the increased occurrence of carotid atherosclerosis [25] and coronary artery calcification progression as evaluated by the coronary artery calcification score [26]. On the other hand, some studies have not demonstrated an association between the TyG index and cardiovascular events. Laura et al. did not find an association between the TyG index and CVD in subjects with T2DM or hypertension at baseline [27]. Cho et al. did not find an independent association between the TyG index and coronary artery disease (CAD) patients with diabetes after adjusting for traditional cardiovascular risk factors [28].

This disparity of these results could be due to differences in participant selection, in event definition or in the research. Moreover, these results could be explained by the hypothesis that patients previously diagnosed with hyperglycemia or hypertriglyceridemia were subjected to targeted treatments and had adopted healthier habits, so their analytical parameters might be better controlled [29].

In conclusion, although to date it is not known the exact mechanisms of association between the TyG index and MACCEs, the TyG index seems to significantly be an independent cardiovascular risk factor.

The clinical implication of our study is that the TyG index could be used as a simple, inexpensive and easily accessible tool to stratify risk in patients with coronary artery disease and could help clinicians in therapeutic and preventive decisions. Since the TyG index can be obtained from a common routine laboratory test, its implementation in clinical practice could be immediate, especially in limited-resource settings. Furthermore, the TyG index is much more practical and accessible than the HOMA-IR index and, in our study, it was more sensitive in predicting cardiovascular events than the HOMA-IR index.

The data emerging from our research have highlighted that, as is known, optimal control of cardiovascular risk factors is a fundamental pillar in the management of patients in both primary and secondary prevention. Therefore, in addition to traditional reference targets such as LDL-C, a new parameter such as the TyG index should be taken into consideration for a more precise risk stratification. Furthermore, since the TyG index, in our study, has shown an important prognostic role in predicting future cardio and cerebrovascular events in ACS patients, it follows that in the post-ACS period, measures should be implemented to reduce the TyG index values, aiming at a reduction in triglycerides and fasting blood glucose.

In fact, the present study still requires improvement. The progression of cardiovascular and cerebrovascular complications in patients at high cardiovascular risk is dynamic and long term, and the inclusion of only baseline data in the analyses may introduce bias into the results. Further investigations into larger patient cohorts are needed to further validate our findings.

### Study Limitations

This study has some limitations.

The first is the small number of patients considered, which implies that bigger prospective studies are needed to confirm our analysis. Second, FPG and triglyceride levels were only measured at the baseline. It follows that if triglyceride values changed during follow-up they could have influenced cardiovascular events. Third, in our study, the association between the TyG index and the Synergy Between PCI With Taxus and Cardiac Surgery (SYNTAX) score was not evaluated. Finally, in our study, the prognostic role of the TyG index was not compared with other biomarkers that provide prognostic information such as high-sensitivity cardiac troponin or against the natriuretic peptides; we intend to evaluate this in the next research.

## Figures and Tables

**Table 1:** Baseline clinical characteristics and clinical events data. Univariate analysis. ACEi, angiotensin-Converting Enzyme Inhibitors; ARB, angiotensin II receptor blockers; CKD-EPI, Chronic Kidney Disease Epidemiology Collaboration; C-reactive protein, ESR erythrocyte sedimentation rate; FPG, fasting plasma glucose; GFR, glomerular filtration rate; HbA1c, glycated haemoglobin; HDL-C, high-density lipoprotein cholesterol; HOMA-IR, homeostasis model assessment-insulin resistance; hs-CRP, high-sensitivity; IFG, impaired fasting glucose; IGT, impaired glucose tolerance; IMT, intima-media thickness; LDL-C, low-density lipoprotein cholesterol; LVEF, left ventricle ejection fraction; NGT, normal glucose tolerance; NSTEMI, non-ST-elevation myocardial infarction; PPG, post-prandial glucose; STEMI, ST-elevation myocardial infarction; TyG index, triglyceride-glucose index; UAP, unstable angina pectoris.

	Tertile 1 (n = 37)	Tertile 2 (n = 39)	Tertile 3 (n = 39)	*p* Value < 0.05
Mean age (n/SD)	67.94 ± 14.16	64.97 ± 11.01	64.35 ± 11.23	0.396
Male *n* (%)	23 (62.12%)	29 (74.35%)	29 (74.35%)	0.408
Current smokers	12(32.43.20%)	16 (41.02%)	11 (28.20%)	0.476
Ex smokers	11 (29.72%)	18 (46.15%)	16 (41.02%)	0.326
Hypertension	28(75.67%)	30 (46.15%)	29 (74.35%)	0.890
Diabetes	11 (29.72%)	18 (76.92%)	28 (71.79%)	0.001
Hypercholesterolaemia	29 (78.37%)	31 (79.48%)	32 (82.05%)	0.680
Body mass index. kg/m^2^	26.73 ± 3.14	28.06 ± 4.73	29.05 ± 4.51	0.238
NGT	16 (43.24%)	15 (40.54%)	4 (10.25%)	0.002
IFG	3 (8.10%)	4 (10.81%)	3 (7.69%)	0.888
IGT	6 (16.21%)	2 (5.40%)	0 (0%)	0.015
**Clinical presentation**				
NSTEMI	11 (29.72%)	10 (25.64%)	9 (23.07%)	0.510
STEMI	12 (32.43%)	12 (30.76%)	11 (28.20%)	0.793
UAP	14 (37.83%)	17 (43.58%)	19 (48.71%)	0.632
Number of vessels treated	1.65 ± 0.84	2 ± 0.93	2.03 ± 0.92	0.167
**Laboratory findings**				
Creatinine	1.05 ± 0.25	1.28 ± 0.44	1.31 ± 0.36	0.020
GFR (CKD-EPI Equations) mL/min/1.73 m^2^	73.90 ± 19.23	64.04 ± 22.96	65.29 ± 23.37	0.229
IMT > 1.5 mm	9 (24.32%)	5 (12.82%)	7 (17.94%)	0.371
Previous cardiovascular events	10 (27.02%)	14 (35.89%)	17 (43.58%)	0.1321
Total cholesterol. mg/dL	160.64 ± 44.19	179.53 ± 57.71	169.41 ± 43.79	0.248
LDL-C. mg/dL	97.69 ± 38.74	111.34 ± 53.51	91.51 ± 35.26	0.129
HDL-C. mg/dL	45.02 ± 11.48	43.15 ± 14.78	38.39 ± 11.90	0.075
Triglycerides. mg/dL	85.35 ± 21.27	130.38 ± 33.11	197.10 ± 68.17	<0.001
Hs-CRP. mg/dL	1.21 ± 1.23	1.54 ± 1.86	1.99 ± 3.09	0.326
ESR	17.38 ± 14.47	19.75 ± 16.97	20.21 ± 20.56	0.791
Mean TyG index	4.51 ± 0.15	4.95 ± 0.07	5.30 ± 0.12	<0.001
Uric acid	5.63 ± 2.46	5.57 ± 1.55	5.71 ± 1.96	0.951
Fibrinogen	374.45 ± 85.29	360.53 ± 84.28	393.48 ± 116.07	0.324
TroponinI	10.85 ± 23.23	11.25 ± 19.70	11.05 ± 24.37	0.997
FPG	102.43 ± 24.99	119.84 ± 38.16	162.02 ± 49.19	<0.001
PPG	127.48 ± 45.19	124.48 ± 40.35	156.37 ± 50.54	0.006
Fasting insulin	8.32 ± 5.84	13.15 ± 14.20	11.55 ± 12.55	0.184
Post-prandial insulin	28.10 ± 22.02	34.61 ± 37.40	20.13 ± 13.54	0.006
HOMA-IR	3.06 ± 1.41	4.68 ± 3.36	5.93 ± 6.2	0.032
HbAlc	5.83 ± 0.43	7 ± 1.19	7.3 ± 1.87	0.327
**Echocardiographic findings**				
LVEF < 40%	1 (2.7%)	0(%)	0 (0%)	0.20
LVEF 40–49%	4 (10.81%)	5 (12.82%)	3 (7.69%)	0.890
LVEF ≥ 50%	32 (86.48)	34 (87.17)	36 (92.30)	0.917
**Medications at discharge**				
Aspirin	37 (100%)	39 (100%)	39 (100%)	0.9658
Clopidogrel/Ticagrelor	32 (86.48%)	34 (87.17%)	32 (82.05%)	0.5438
β-blocker	28 (75.67%)	30 (76.92%)	35 (89.74%)	0.1121
ACEI/ARB	32 (86.48%)	29 (74.35%)	32 (82.05%)	0.6117
Statin	28 (75.67%)	30 (76.92%)	31 (79.48%)	0.6887
CCB	5 (13.51%)	4 (10.25%)	3 (7.69%)	0.4114
Nitrate	7 (18.91%)	7 (17.94%)	4 (10.25%)	0.2968
Insulin	10 (27.02%)	14 (35.89%)	17 (43.58%)	0.1321

**Table 2:** MACCEs’ major adverse cardiovascular and cerebrovascular events. Univariate analysis.

	Tertile 1 (n = 37)	Tertile 2 (n = 39)	Tertile 3 (n = 39)	*p* Value < 0.05
**MACCEs**	12 (32.43%)	22 (56.41%)	29 (74.35%)	0.0002
**Unstable angina pectoris**	5 (13.51%)	11 (28.20%)	15 (38.46%)	0.0144
**Acute myocardial infarction**	1 (2.70%)	2 (5.12%)	6 (15.38%)	0.0385
**Heart failure**	4 (10.81%)	3 (7.69%)	4 (5.12%)	0.9098
**Cardiac arrest**	2 (5.40%)	5 (12.82%)	2 (5.12%)	0.9467
**Coronary revascularization**	4 (10.81%)	7 (17.94%)	8 (20.51%)	0.2569
**Stroke**	0 (0%)	1 (2.56%)	2 (5.12%)	0.1609

## Data Availability

The datasets used in the study are available from the corresponding author upon reasonable request.
